# Trends and Disparities in Liver Cancer Incidence by Demographic Factors in the United States (1999-2020)

**DOI:** 10.7759/cureus.95552

**Published:** 2025-10-28

**Authors:** Karine Vartanian, Laxmi Mahita Reddy Paripati, Mohammed Sohail Parvez, Akshitha Talasila, Sruti Mohanty, Surbhi Dadwal, Rajmohan Seetharaman

**Affiliations:** 1 Cardiology, Southern California Hospital Heart Institute, Culver City, USA; 2 Internal Medicine, Malla Reddy Institute of Medical Sciences, Hyderabad, IND; 3 Surgery, Deccan College of Medical Sciences, Hyderabad, IND; 4 Surgery, Mamata Academy of Medical Sciences, Hyderabad, IND; 5 Medicine, Srirama Chandra Bhanja (SCB) Medical College and Hospital, Cuttack, IND; 6 Oncology, Dr. Rajendra Prasad Government Medical College, Kangra, IND; 7 Pharmacology, Mahatma Gandhi Mission (MGM) Medical College and Hospital, MGM Institute of Health Sciences, Navi Mumbai, IND

**Keywords:** cancer, cdc-wonder, disparity, incidence, liver

## Abstract

Introduction: Analyzing liver cancer incidence is essential for identifying trends and determining risk factors, which, in turn, guide strategies for prevention and treatment. By focusing on high-risk populations, such analyses enhance health outcomes and cancer risk management.

Methodology: A retrospective study was conducted using data from the Centers for Disease Control-Wide-ranging Online Data for Epidemiologic Research database (1999-2020) to evaluate liver cancer incidence trends in the United States (US) by age, gender, and race.

Results: Demographic variations of liver cancer in the US from 1999 to 2020 were determined. A state-wise distribution of crude rates per 100,000 and incidence across the US during the same period was established. Based on age, gender, and race, the fluctuating trends in liver cancer incidence from 1999 to 2020 were also investigated.

Conclusions: This study shows state-wise variations in liver cancer incidence; however, crude rates per 100,000 remain relatively consistent across states. Over 22 years, liver cancer rates have increased, with the highest incidence found in men, White individuals, and those aged 65-74. Incidence increased for individuals over 45, but by the end of two decades, rates for ages 45-54 decreased while remaining high for ages ≥55. A more prominent upward trajectory was observed in men compared to women and in Whites compared to other races, underscoring the need for targeted prevention and control strategies.

## Introduction

Liver cancer is a leading cause of cancer-related deaths worldwide, the fifth most common cancer in the United States (US), and one of the deadliest, with annual incidence continuing to rise [1]. Mortality rates for primary liver cancer have increased more rapidly than for any other major cancer type. In the US, hepatocellular carcinoma accounts for approximately 90% of primary liver cancer cases, while intrahepatic cholangiocarcinoma represents most of the remaining 10% [2,3].

Globally, a direct correlation is observed between hepatocellular carcinoma incidence and age, up to around 75 years. However, the median age at diagnosis is slightly younger. In the US, the median age at diagnosis is 60-64 years for men and 65-69 years for women. Incidence rates in men are typically two to four times higher than in women [4]. In 2016, the age-adjusted incidence rate was 10.4 per 100,000 for men and 2.9 per 100,000 for women [4]. In multiethnic societies like the US, racial and ethnic disparities are particularly significant [4].

Geographic, age, sex, and racial variations in incidence primarily reflect differences in exposure to risk factors, though additional determinants may contribute [4]. Major risk factors include chronic hepatitis B and C virus infections, excessive alcohol consumption, and metabolic conditions such as type 2 diabetes, obesity, metabolic syndrome, and nonalcoholic fatty liver disease [4,5]. Other contributors include cigarette smoking, ingestion of aflatoxin-contaminated foods, liver fluke infestation, and genetic predisposition [4,5].

This study is important for characterizing disease patterns across populations and regions. Identifying high-risk groups can guide prevention strategies and help predict the future burden of liver cancer. Given its status as a leading cause of cancer mortality, continued research in this area remains vital for public health. This study aimed to assess liver cancer incidence in the US from 1999 to 2020 according to demographic variables, including age, gender, and race.

## Materials and methods

This is a retrospective original study that was conducted using the Centers for Disease Control-Wide-ranging Online Data for Epidemiologic Research (CDC-WONDER). The CDC-WONDER system is an online database for public health data provided by the CDC [6]. As this is a nonhuman participant research and since the CDC-WONDER website contains deidentified data, the approval of the ethics committee was not required.

The data from CDC-WONDER were collected on a single day, May 18, 2024. The dataset that matches the research was chosen, as, in this research, it was cancer statistics data: “cancer statistics were selected (all causes).” Cancer incidence from 1999 to 2020 was chosen. Then, the site of cancer was selected, i.e., liver cancer, and crude rates for liver cancer were chosen. For temporal trends, the year was selected, and for demography, age, gender, and race were chosen. The data were collected by year and stratified by age, gender, and race to identify trends over time in accordance with the states in which the disease is prevalent.

The data were exported to a Microsoft Excel sheet (Microsoft Corporation, Redmond, WA), and statistical analysis was conducted using R version 4.3.1 (R Foundation for Statistical Computing, Vienna, Austria). The figures/graphs were created using ggplot2, version 3.5.0 (R Foundation for Statistical Computing, Vienna, Austria).

## Results

From 1999 to 2020, the incidence of liver cancer in the US was 513,970 in a total population of 347,675,258. The crude rate per 100,000 was 7.28. Table 1 shows the demographic characteristics of liver cancer patients in the US from 1999 to 2020 based on age, gender, and race. The crude rate per 100,000 was highest in the age group ≥75 years (crude rate 26.75), men (crude rate 10.92), and Asian/Pacific Islanders (crude rate 10.12).

**Table 1:** Demographic characteristics of liver cancer patients in United States from 1999-2020 based on age, gender, and race The mortality is mentioned in n (%) format, and the crude rate is mentioned per 100,000 population

Parameter	Population	Count, n (%)	Crude rate per 100,000
Age (years)			
<15	1,393,049,191	4,323 (0.84%)	0.31
15-24	984,207,074	1,526 (0.3%)	0.16
25-34	959,283,218	3,097 (0.6%)	0.32
35-44	973,812,431	10,754 (2.09%)	1.1
45-54	968,241,074	69,160 (13.46%)	7.14
55-64	808,129,415	166,022 (32.3%)	20.54
65-74	540,456,270	142,565 (27.74%)	26.38
≥75	435,628,239	116,523 (22.67%)	26.75
Gender			
Male	3,481,479,919	380,110 (73.96%)	10.92
Female	3,581,326,993	133,860 (26.04%)	3.74
Race			
American Indian or Alaskan Native	93,121,600	6,176 (1.2%)	6.63
Asian or Pacific Islander	398,633,095	40,347 (7.85%)	10.12
Black or African American	964,214,883	74,964 (14.59%)	7.77
White	5,606,837,334	388,151 (75.52%)	6.92
Other races	Not applicable	4,332 (0.84%)	Not applicable

**Figure 1:**
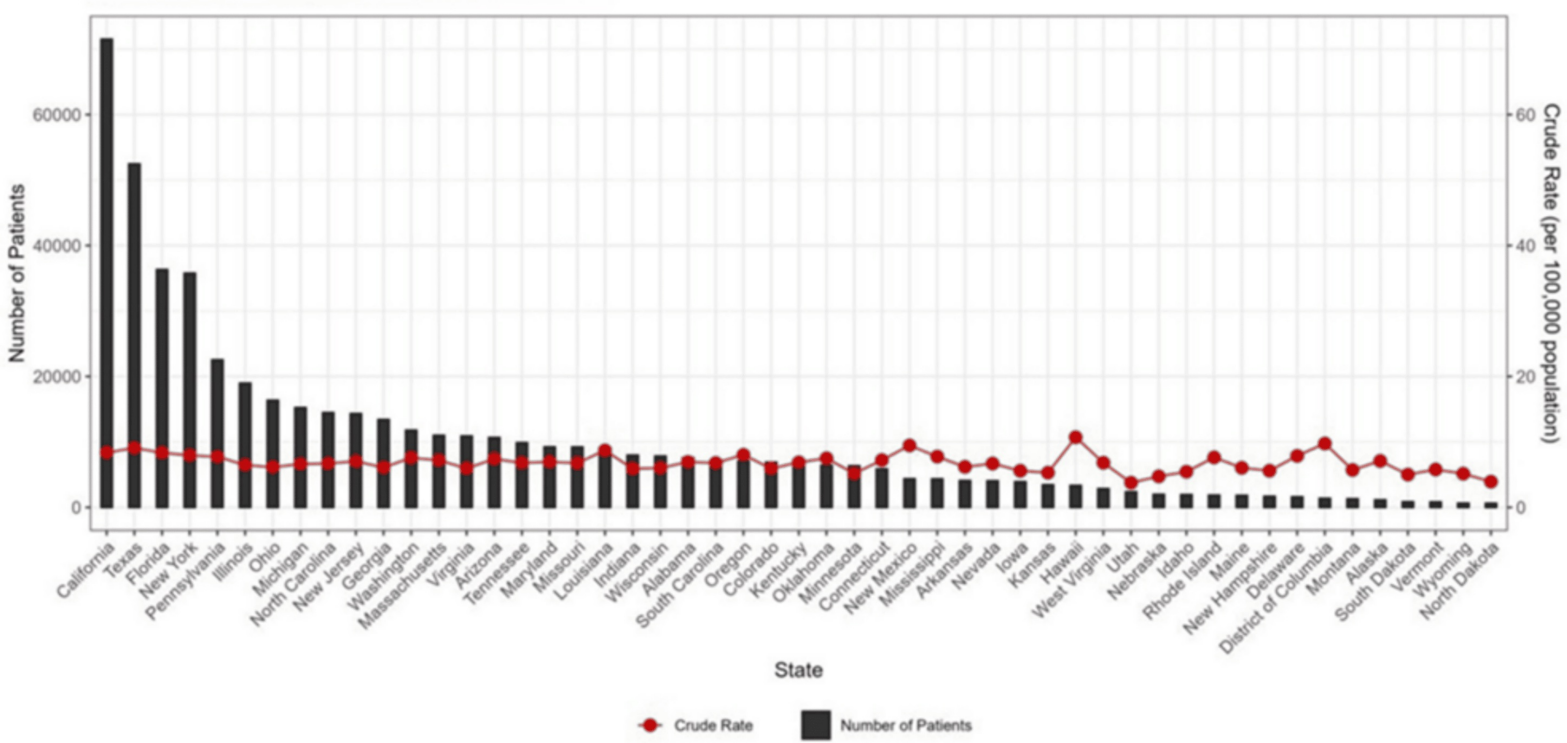
State-wise distribution of liver cancer patients in United States from 1999 to 2020

Figure 1 shows the state-wise distribution of liver cancer patients in the US from 1999 to 2020. In the last 21 years, the incidence of liver cancer in the US was highest in California, followed by Texas. It was the lowest in North Dakota.

**Figure 2:**
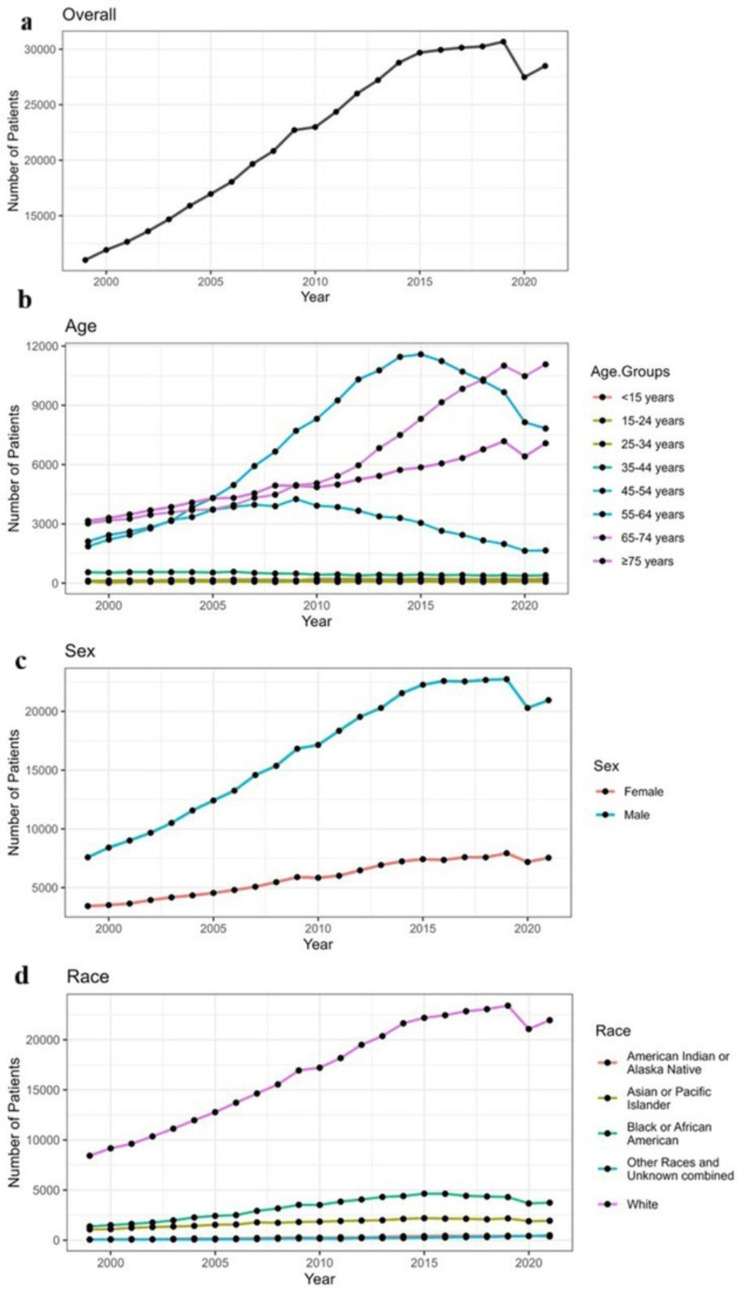
Temporal trends in the incidence of liver cancer in United States from 1999 to 2020 based on age, gender, and race (a) Overall: the total number of patients per year. There is a general upward trend from 1999 to 2019, with a slight decline around 2020 likely reflecting pandemic-related disruptions. (b) By age group: the number of patients stratified by age groups. Patients aged 45-64 and 65-74 years show the most notable increases, particularly after 2010, while younger age groups remain relatively stable or decline. (c) By sex: trends separated by male and female patients. Male patients consistently account for a higher number of patients than female patients throughout the study period. (d) By race: the number of patients by racial groups. White patients comprise the largest share, with notable increases over time. Other racial groups show relatively modest growth

Figure 2 shows temporal trends in the incidence of liver cancer in the US from 1999 to 2020 based on age, gender, and race. The incidence of liver cancer in the US has been rising from 1999 to 2020; the incidence was the highest in 2019. The incidence of liver cancer has been steadily rising in age groups 55-64 and has declined in age groups 45-54. The incidence of liver cancer in men has been rising, and so has the incidence in women. But the rise in the incidence of liver cancer in men is much higher (almost threefold) than that observed in women. The incidence of liver cancer in Whites is increasing exponentially, whereas the incidence in American Indian/Alaska Native and other races and unknown combined has been less and stable.

## Discussion

A retrospective research study was conducted to assess the incidence of liver cancer in the US over 22 years. Data were collected from the CDC-WONDER database. This study revealed that out of the 7,062,806,912 population analyzed, the crude rate of liver cancer was 7.28 per 100,000 over 20 years. The incidence was highest in the age group >75 years (crude rate 26.75), men (crude rate 10.92), and Asian/Pacific Islanders (crude rate 10.12). The temporal trends show the crude rate of liver cancer rising, with a slight fall by the end of the period (Figure 2).

According to the World Cancer Research Fund International data, more than 866,136 new cases of liver cancer were diagnosed in 2022 worldwide. The US ranks second in the global rank of liver cancer incidence in 2022, with 43,492 new cancer cases and a 6.8 per 100,000 crude rate. The highest incidence of liver cancer was in the population of China, with 367,677 cases [7]. Despite being relatively rare, compared to other cancers (2.1% of all new cancer cases in the US), liver and intrahepatic bile duct cancer is the sixth leading cause of cancer death in the US, with a 21.7% five-year relative survival rate [8]. In the US, primary liver cancer has become the most rapidly growing cancer in terms of incidence, in both men and women. From 2012 to 2016, the incidence of liver cancer increased by 2.5%, the largest increase of any cancer during that period, and death rates for this disease have more than doubled since 1980 [9,10]. Such growth in liver cancer incidence is likely explained by the exponential rise in risk factors within the American population, including hepatitis B and C, alcohol use disorder with subsequent cirrhosis, diabetes, and obesity, with the development of nonalcoholic steatohepatitis (NASH). An additional contributing factor is the increasing number of elderly individuals in the population.

Age-based analysis of liver cancer incidence in the present study showed the highest incidence in individuals over 75 years. Genomic instability, telomere attrition, and genetic alterations may play a crucial role in hepatocarcinogenesis [11,12].

In most countries, the incidence rate of liver cancer among men is higher than among women, with male-to-female ratios averaging between 2:1 and 4:1 [13]. According to a study, the crude rate of liver cancer in men reached 10.92 compared to 3.74 in women, which aligns with most of the studies in the US and European countries [4,13]. The higher rates of liver cancer in men are likely related to higher exposure to risk factors, increased iron stores, and androgen influence [4].

The statistics of this study showed that Asian or Pacific Islanders had the highest crude incidence rate of 10.12 per 100,000. In contrast, American Indians or Alaskan Natives had the lowest crude rate of 6.63 per 100,000. These findings align with studies by Petrick et al., with a similar trend of increased incidence of hepatocellular carcinoma among the Asian/Pacific Islanders [14].

Liver cancer is frequently diagnosed in an advanced stage; therefore, screening, early detection of the disease, and preventive measures are crucial in further improvement of a patient's survival rate. Global guidelines recommend surveillance of high-risk populations like patients with cirrhosis, hepatitis B, and hepatitis C, which include abdominal ultrasound and serum alpha-fetoprotein (AFP) measurement every six months. Further assessment with dynamic contrast-enhanced computed tomography and multimodal magnetic resonance imaging scans is required in patients with a positive screening result (mass >1 cm on ultrasonography or AFP level >20 ng/m) [15,16]. Preventive strategies to minimize risk factor exposure include vaccination against hepatitis B, abstention from smoking and excessive alcohol consumption, avoiding foods containing aflatoxin B1 (e.g., nuts, corn), and maintaining a healthy weight.

This study has certain limitations. Due to the COVID-19 pandemic, the latest data from 2021 to 2023 was not included in the study, which affected data collection. Such details as stage, grade, and outcome of liver cancer could not be collected.

## Conclusions

The rising incidence of liver cancer highlights the need for increased awareness and education about the disease. By understanding the risk factors and trends, healthcare professionals can work toward reducing the burden of liver cancer and improving patient outcomes. Effective prevention and treatment strategies can help mitigate the impact of liver cancer on individuals and communities. By prioritizing liver cancer research and education, we can work toward reducing the incidence and mortality rates associated with this disease. Ultimately, a comprehensive approach to liver cancer prevention and treatment can improve the lives of those affected and save lives. Future research should focus on identifying emerging risk factors, evaluating disparities across demographic groups, and developing targeted interventions for high-risk populations.
